# Supplementary material for: OpenMM 7: Rapid development of high performance algorithms for molecular dynamics
Source: PLoS Comput Biol. 2017 Jul 26;13(7):e1005659. doi: 10.1371/journal.pcbi.1005659 (PMC5549999; doi:10.1371/journal.pcbi.1005659)
Supplement: S1 Source Code — (ZIP) [file pcbi.1005659.s002.zip › OpenMM-7.0.1-Source/docs/api-python/generated/simtk.openmm.openmm.Context.html]

Context — OpenMM 7.0.1.dev-5e86c4f documentation


# Context¶

*class* `simtk.openmm.openmm.``Context`(*\*args*)¶
:   A Context stores the complete state of a simulation. More specifically, it includes:

    > - The current time
    > - The position of each particle
    > - The velocity of each particle
    > - The values of configurable parameters defined by Force objects in the System

    You can retrieve a snapshot of the current state at any time by calling getState(). This allows you to record the state of the simulation at various points, either for analysis or for checkpointing. getState() can also be used to retrieve the current forces on each particle and the current energy of the System.

    `__init__`(*self*, *system*, *integrator*) → Context¶
    :   \_\_init\_\_(self, system, integrator, platform) -> Context
        \_\_init\_\_(self, system, integrator, platform, properties) -> Context
        \_\_init\_\_(self, other) -> Context

        Construct a new Context in which to run a simulation, explicitly specifying what Platform should be used to perform calculations and the values of platform-specific properties.

        |  |  |
        | --- | --- |
        | Parameters: | - **system** (*System*) – the System which will be simulated - **integrator** (*Integrator*) – the Integrator which will be used to simulate the System - **platform** (*Platform*) – the Platform to use for calculations - **properties** (*map< std::string, std::string >*) – a set of values for platform-specific properties. Keys are the property names. |

    Methods

    |  |  |
    | --- | --- |
    | `__init__`((self, system, integrator) -> Context) | \_\_init\_\_(self, system, integrator, platform) -> Context |
    | `applyConstraints`(self, tol) | Update the positions of particles so that all distance constraints are satisfied. |
    | `applyVelocityConstraints`(self, tol) | Update the velocities of particles so the net velocity of each constrained distance is zero. |
    | `computeVirtualSites`(self) | Recompute the locations of all virtual sites. |
    | `createCheckpoint`((self) -> std::string) | Create a checkpoint recording the current state of the Context. |
    | `getIntegrator`((self) -> Integrator) | getIntegrator(self) -> Integrator |
    | `getMolecules`((self) -> vectorii) | Get a description of how the particles in the system are grouped into molecules. |
    | `getParameter`((self, name) -> double) | Get the value of an adjustable parameter defined by a Force object in the System. |
    | `getParameters`((self) -> mapstringdouble) | Get all adjustable parameters that have been defined by Force objects in the System, along with their current values. |
    | `getPlatform`((self) -> Platform) | getPlatform(self) -> Platform |
    | `getState`([getPositions, getVelocities, ...]) | Get a State object recording the current state information stored in this context. |
    | `getSystem`((self) -> System) | Get System being simulated in this context. |
    | `loadCheckpoint`(self, checkpoint) | Load a checkpoint that was written by createCheckpoint(). |
    | `reinitialize`(self) | When a Context is created, it may cache information about the System being simulated and the Force objects contained in it. |
    | `setParameter`(self, name, value) | Set the value of an adjustable parameter defined by a Force object in the System. |
    | `setPeriodicBoxVectors`(self, a, b, c) | Set the vectors defining the axes of the periodic box (measured in nm). |
    | `setPositions`(self, positions) | Set the positions of all particles in the System (measured in nm). |
    | `setState`(Context self, State state) | Copy information from a State object into this Context. |
    | `setTime`(self, time) | Set the current time of the simulation (in picoseconds). |
    | `setVelocities`(self, velocities) | Set the velocities of all particles in the System (measured in nm/picosecond). |
    | `setVelocitiesToTemperature`(self, ...) | setVelocitiesToTemperature(self, temperature) |

    `getSystem`(*self*) → System¶
    :   Get System being simulated in this context.

    `getIntegrator`(*self*) → Integrator¶
    :   getIntegrator(self) -> Integrator

        Get Integrator being used to by this context.

    `getPlatform`(*self*) → Platform¶
    :   getPlatform(self) -> Platform

        Get the Platform being used for calculations.

    `setTime`(*self*, *time*)¶
    :   Set the current time of the simulation (in picoseconds).

    `setPositions`(*self*, *positions*)¶
    :   Set the positions of all particles in the System (measured in nm). This method simply sets the positions without checking to see whether they satisfy distance constraints. If you want constraints to be enforced, call applyConstraints() after setting the positions.

        |  |  |
        | --- | --- |
        | Parameters: | **positions** (*vector< Vec3 >*) – a vector whose length equals the number of particles in the System. The i’th element contains the position of the i’th particle. |

    `setVelocities`(*self*, *velocities*)¶
    :   Set the velocities of all particles in the System (measured in nm/picosecond).

        |  |  |
        | --- | --- |
        | Parameters: | **velocities** (*vector< Vec3 >*) – a vector whose length equals the number of particles in the System. The i’th element contains the velocity of the i’th particle. |

    `setVelocitiesToTemperature`(*self*, *temperature*, *randomSeed*)¶
    :   setVelocitiesToTemperature(self, temperature)

        Set the velocities of all particles in the System to random values chosen from a Boltzmann distribution at a given temperature.

        |  |  |
        | --- | --- |
        | Parameters: | - **temperature** (*double*) – the temperature for which to select the velocities (measured in Kelvin) - **randomSeed** (*int*) – the random number seed to use when selecting velocities |

    `getParameters`(*self*) → mapstringdouble¶
    :   Get all adjustable parameters that have been defined by Force objects in the System, along with their current values.

    `getParameter`(*self*, *name*) → double¶
    :   Get the value of an adjustable parameter defined by a Force object in the System.

        |  |  |
        | --- | --- |
        | Parameters: | **name** (*string*) – the name of the parameter to get |

    `setParameter`(*self*, *name*, *value*)¶
    :   Set the value of an adjustable parameter defined by a Force object in the System.

        |  |  |
        | --- | --- |
        | Parameters: | - **name** (*string*) – the name of the parameter to set - **value** (*double*) – the value of the parameter |

    `setPeriodicBoxVectors`(*self*, *a*, *b*, *c*)¶
    :   Set the vectors defining the axes of the periodic box (measured in nm). They will affect any Force that uses periodic boundary conditions.

        Triclinic boxes are supported, but the vectors must satisfy certain requirements. In particular, a must point in the x direction, b must point “mostly” in the y direction, and c must point “mostly” in the z direction. See the documentation for details.

        |  |  |
        | --- | --- |
        | Parameters: | - **a** (*Vec3*) – the vector defining the first edge of the periodic box - **b** (*Vec3*) – the vector defining the second edge of the periodic box - **c** (*Vec3*) – the vector defining the third edge of the periodic box |

    `applyConstraints`(*self*, *tol*)¶
    :   Update the positions of particles so that all distance constraints are satisfied. This also recomputes the locations of all virtual sites.

        |  |  |
        | --- | --- |
        | Parameters: | **tol** (*double*) – the distance tolerance within which constraints must be satisfied. |

    `applyVelocityConstraints`(*self*, *tol*)¶
    :   Update the velocities of particles so the net velocity of each constrained distance is zero.

        |  |  |
        | --- | --- |
        | Parameters: | **tol** (*double*) – the velocity tolerance within which constraints must be satisfied. |

    `computeVirtualSites`(*self*)¶
    :   Recompute the locations of all virtual sites. There is rarely a reason to call this, since virtual sites are also updated by applyConstraints(). This is only for the rare situations when you want to enforce virtual sites but <i>not</i> constraints.

    `reinitialize`(*self*)¶
    :   When a Context is created, it may cache information about the System being simulated and the Force objects contained in it. This means that, if the System or Forces are then modified, the Context might not see all of the changes. Call reinitialize() to force the Context to rebuild its internal representation of the System and pick up any changes that have been made.

        This is an expensive operation, so you should try to avoid calling it too frequently.

    `getMolecules`(*self*) → vectorii¶
    :   Get a description of how the particles in the system are grouped into molecules. Two particles are in the same molecule if they are connected by constraints or bonds, where every Force object can define bonds in whatever way are appropriate to that force.

        Each element lists the indices of all particles in a single molecule. Every particle is guaranteed to belong to exactly one molecule.

    `getState`(*getPositions=False*, *getVelocities=False*, *getForces=False*, *getEnergy=False*, *getParameters=False*, *enforcePeriodicBox=False*, *groups=-1*)¶
    :   Get a State object recording the current state information stored in this context.

        |  |  |
        | --- | --- |
        | Parameters: | - **getPositions** (*bool=False*) – whether to store particle positions in the State - **getVelocities** (*bool=False*) – whether to store particle velocities in the State - **getForces** (*bool=False*) – whether to store the forces acting on particles in the State - **getEnergy** (*bool=False*) – whether to store potential and kinetic energy in the State - **getParameter** (*bool=False*) – whether to store context parameters in the State - **enforcePeriodicBox** (*bool=False*) – if false, the position of each particle will be whatever position   is stored in the Context, regardless of periodic boundary conditions.   If true, particle positions will be translated so the center of   every molecule lies in the same periodic box. - **groups** (*set={0,1,2,...,31}*) – a set of indices for which force groups to include when computing   forces and energies. The default value includes all groups. groups   can also be passed as an unsigned integer interpreted as a bitmask,   in which case group i will be included if (groups&(1<<i)) != 0. |

    `setState`(*Context self*, *State state*)¶
    :   Copy information from a State object into this Context. This restores the Context to
        approximately the same state it was in when the State was created. If the State does not include
        a piece of information (e.g. positions or velocities), that aspect of the Context is
        left unchanged.

        Even when all possible information is included in the State, the effect of calling this method
        is still less complete than loadCheckpoint(). For example, it does not restore the internal
        states of random number generators. On the other hand, it has the advantage of not being hardware
        specific.

    `createCheckpoint`(*self*) → std::string¶
    :   Create a checkpoint recording the current state of the Context.
        This should be treated as an opaque block of binary data. See loadCheckpoint() for more details.

        Returns: a string containing the checkpoint data

    `loadCheckpoint`(*self*, *checkpoint*)¶
    :   Load a checkpoint that was written by createCheckpoint().

        A checkpoint contains not only publicly visible data such as the particle positions and
        velocities, but also internal data such as the states of random number generators. Ideally,
        loading a checkpoint should restore the Context to an identical state to when it was written,
        such that continuing the simulation will produce an identical trajectory. This is not strictly
        guaranteed to be true, however, and should not be relied on. For most purposes, however, the
        internal state should be close enough to be reasonably considered equivalent.

        A checkpoint contains data that is highly specific to the Context from which it was created.
        It depends on the details of the System, the Platform being used, and the hardware and software
        of the computer it was created on. If you try to load it on a computer with different hardware,
        or for a System that is different in any way, loading is likely to fail. Checkpoints created
        with different versions of OpenMM are also often incompatible. If a checkpoint cannot be loaded,
        that is signaled by throwing an exception.

        Parameters:
        :   - checkpoint (string) the checkpoint data to load

    `__delattr__`¶
    :   x.\_\_delattr\_\_(‘name’) <==> del x.name

    `__format__`()¶
    :   default object formatter

    `__getattribute__`¶
    :   x.\_\_getattribute\_\_(‘name’) <==> x.name

    `__hash__`¶

    `__reduce__`()¶
    :   helper for pickle

    `__reduce_ex__`()¶
    :   helper for pickle

    `__sizeof__`() → int¶
    :   size of object in memory, in bytes

    `__str__`¶

High performance molecular simulation on GPUs


### Navigation

- Application Layer
- Library Layer
  - Core Objects
  - Forces
  - Integrators
  - Extras

©2015, Stanford University and the Authors.
|
Powered by Sphinx 1.3.1
& Alabaster 0.7.7
|
Page source
